# At the tip of an iceberg: citizen science and active surveillance collaborating to broaden the known distribution of *Aedes japonicus* in Spain

**DOI:** 10.1186/s13071-021-04874-4

**Published:** 2021-07-26

**Authors:** Roger Eritja, Sarah Delacour-Estrella, Ignacio Ruiz-Arrondo, Mikel A. González, Carlos Barceló, Ana L. García-Pérez, Javier Lucientes, Miguel Á. Miranda, Frederic Bartumeus

**Affiliations:** 1 Centre de Recerca Ecològica i Aplicacions Forestals (CREAF), Cerdanyola del Vallès, Barcelona, Spain; 2 The Agrifood Institute of Aragón (IA2), Faculty of Veterinary Medicine, Zaragoza, Spain; 3 Center for Rickettsioses and Arthropod-Borne Diseases, Hospital Universitario San Pedro–CIBIR, Logroño, Spain; 4 NEIKER-Basque Institute for Agricultural Research and Development, Basque Research and Technology Alliance (BRTA), Derio, Spain; 5 Applied Zoology and Animal Conservation research group, Universitat de les Illes Balears (UIB), Palma, Spain; 6 Agro-Environmental and Water Economics Institute (INAGEA), Palma, Spain; 7 Centre d’Estudis Avançats de Blanes (CEAB-CSIC), Blanes, Spain; 8 Institució Catalana de Recerca i Estudis Avançats (ICREA), Barcelona, Spain

**Keywords:** Asian bush mosquito, Culicidae, West Nile virus, Citizen science, Northern Spain, Cantabria, Basque Country

## Abstract

**Background:**

Active surveillance aimed at the early detection of invasive mosquito species is usually focused on seaports and airports as points of entry, and along road networks as dispersion paths. In a number of cases, however, the first detections of colonizing populations are made by citizens, either because the species has already moved beyond the implemented active surveillance sites or because there is no surveillance in place. This was the case of the first detection in 2018 of the Asian bush mosquito, *Aedes japonicus*, in Asturias (northern Spain) by the citizen science platform Mosquito Alert.

**Methods:**

The collaboration between Mosquito Alert, the Ministry of Health, local authorities and academic researchers resulted in a multi-source surveillance combining active field sampling with broader temporal and spatial citizen-sourced data, resulting in a more flexible and efficient surveillance strategy.

**Results:**

Between 2018 and 2020, the joint efforts of administrative bodies, academic teams and citizen-sourced data led to the discovery of this species in northern regions of Spain such as Cantabria and the Basque Country. This raised the estimated area of occurrence of *Ae. japonicus* from < 900 km^2^ in 2018 to > 7000 km^2^ in 2020.

**Conclusions:**

This population cluster is geographically isolated from any other population in Europe, which raises questions about its origin, path of introduction and dispersal means, while also highlighting the need to enhance surveillance systems by closely combining crowd-sourced surveillance with public health and mosquito control agencies’ efforts, from local to continental scales. This multi-actor approach for surveillance (either passive and active) shows high potential efficiency in the surveillance of other invasive mosquito species, and specifically the major vector *Aedes aegypti* which is already present in some parts of Europe.

**Graphical abstract:**

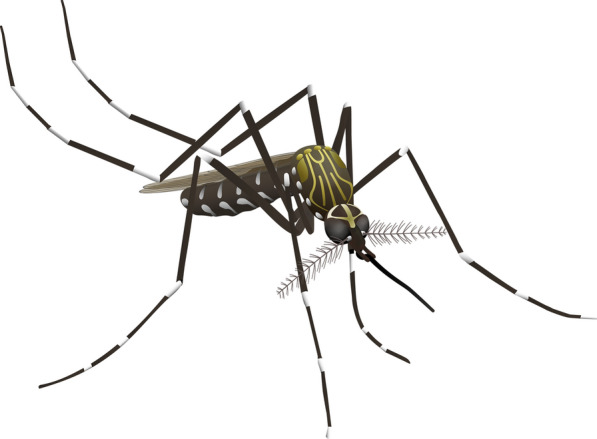

## Background

Since 2002 the invasive Asian bush mosquito *Aedes* (*Hulecoetomyia*) *japonicus* ssp. *japonicus* has been regularly detected in Europe. Populations in different areas are generally assumed to have independently originated from separate colonization events, including plausible transport from the USA and from the species’ original range in Asia, which includes Taiwan, China, Japan, south-eastern Russia and Korea. Similar to *Aedes albopictus*, this species shows relevant invasive abilities [1], based on its adaption to slightly different ecological niches and colonization of colder climate areas both in the USA [2] and in Europe [3]. Whereas in nature *Ae. japonicus* breeds in tree holes and rock pools, it also uses artificial containers, especially used tires [4] and larger containers such as abandoned bathtubs and cattle troughs, accepting moderate loads of organic content in the water [5]. Field evidence confirms that *Ae. japonicus* is mostly mammalophilic, but its real host range is wider since bird-feeding has been also observed [6]. The species usually causes moderate nuisance to humans, especially in the vicinity of deciduous forests. It is active during daytime and evening, and it is mostly exophilic but occasionally enters houses [7]. This species is also able to withstand cold and snowy winters in the form of eggs or larvae [8].

As a vector, *Ae. japonicus* is not considered a high-risk species although field-collected individuals in the USA have been found infected with West Nile virus (WNV) [9] and La Crosse virus (LACV) [10], and laboratory studies have demonstrated an efficiency for WNV transmission higher than that of *Culex pipiens* [11]. Vector competence for other arboviruses, including dengue virus (DENV), Japanese encephalitis virus (JEV) and Rift Valley fever virus (RVFV) has only been verified in laboratory conditions [12, 13]. Despite all these findings, to date there has been no confirmation of the role of *Ae. japonicus* in the field transmission of any of the above-cited arboviruses.

*Aedes japonicus*’ record of invasion worldwide is remarkable, second only to those of *Aedes aegypti* and *Ae. albopictus*. A review of its spread in Europe can be found in Koban et al. [14], which also includes a discussion of surveillance methods. The first detection of *Ae. japonicus* in Europe occurred in 2000 in a French tire depot [7] from which it was eradicated [1]. The species was found later in Belgium in 2002 [15] and in Switzerland in 2008, followed by detection in areas of Germany [1], confirmed in 2009 as widespread in an area of > 10,000 km^2^ [16]. Further monitoring of the expansion across Germany from 2012 to 2015 was guided by the citizen science platform Mueckenatlas, which has made it possible to track the spread of separate populations [17]. *Aedes japonicus* was detected in Austria and Slovenia in 2011 [18]; in 2012, the first records were registered in both the Netherlands [19] and Hungary [18]. Definitive establishment in France [20] as well as in Croatia [21] was confirmed in 2013, with a subsequent spread to Bosnia-Herzegovina and Serbia by the following years [22]. In 2015 the species was found in Italy [18] and Liechtenstein [23] and in 2020 in Romania [24]. Spain was added to the list in 2018 thanks to a notification from a person in the region of Asturias using the citizen science platform Mosquito Alert and subsequent field verification [5]. That study on the first finding in Spain suggested that the introduction was not recent, as the species was already found along a 10-km transect in a rural landscape. The probable colonized area was then estimated at a minimum of 827 km^2^ [5]. Moreover, additional notifications highly compatible with the species were received by Mosquito Alert after a dedicated call to action for further data collection was sent to participants in the region. Based on these data and the isolation of some locations, the hypothesis of a much wider colonized surface was suggested, much as had occurred in the process of discovery along the Swiss–German border [1].

Here, we describe surveillance activities carried out in Spain by regional agencies, the Spanish Ministry of Health and academics from different institutions that had allowed us to gain more knowledge about the distribution of *Ae. japonicus*. Simultaneously and in collaboration with these field operations, Mosquito Alert continued its citizen-based surveillance by encouraging local participants to report mosquitoes—with particular emphasis on uncommon species. Being an internet-based observatory, the confirmation of any report of invasive mosquito species from previously unknown areas of distribution must be performed in the field, and this is done by agreement between Mosquito Alert and the Ministry of Health, as described in the Methods section.

## Methods

**Fig. 1:**
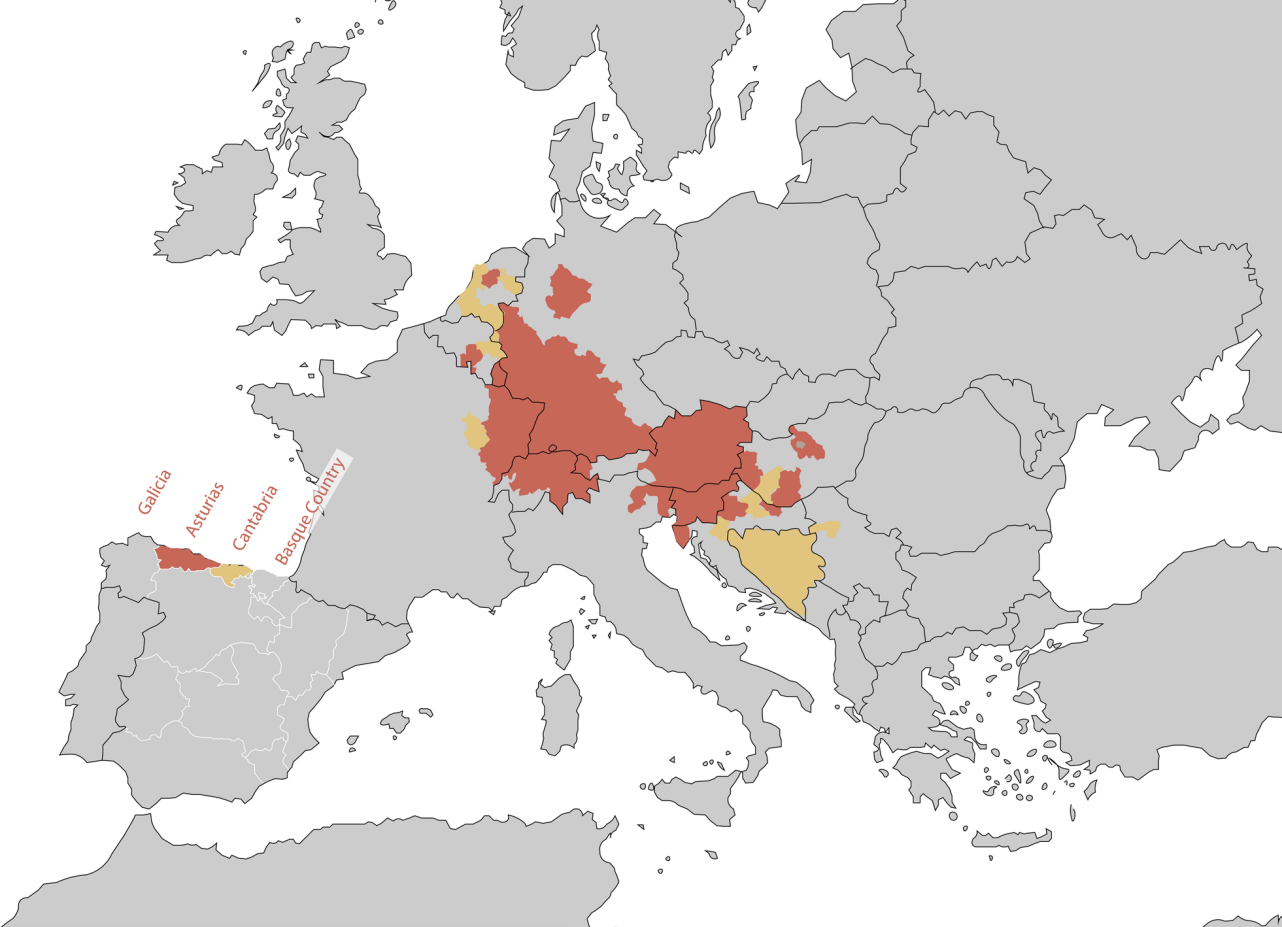
Current distribution status of *Aedes japonicus* in Europe (yellow: introduced; red: established). This area, commonly referred to as ‘Green Spain,’ is suggested as the main natural habitat for *Ae. japonicus* and includes four Autonomous Communities in northern Spain: Principality of Asturias (NUTS ES12), Cantabria (ES13), the Basque Country (ES21) and Galicia (ES11). The detection in Cantabria, described in this work, had already been published in the European Centre for Disease Prevention and Control (ECDC) map following administrative communication. Map is reproduced from the Vectornet/ECDC NUTS2 map of March 2021 [25].

The study area of this work is located in the Cantabrian cornice in northern Spain. At the NUTS2 level, this includes the Autonomous Communities of Galicia, the Principality of Asturias, Cantabria and the Basque Country (Fig. 1). This area is commonly denominated ‘Green Spain’ because its oceanic climate favors a densely vegetated landscape, featuring mild summers and cool, but not cold, winters (mean July and January temperatures: < 20 °C and > 6 °C, respectively), combined with a yearly precipitation level > 1000 mm with precipitation events evenly distributed along the year.

These administrative units have a fair degree of self-governance, including over public health and environmental affairs. This includes the surveillance of invasive mosquito species, which is recommended by the Spanish Government but is not mandatory.

All the monitoring operations in northern Spain discussed in this article were carried out between 2018 and 2020, as described below in detail (items i–v in List). In contrast to Asturias and Cantabria which are single-province autonomous communities, the Basque Country is composed of three provinces (Bizkaia, Gipuzkoa and Araba) which will be discussed separately.

(i) The citizen science platform Mosquito Alert has been permanently active since 2014 under the management of leading research institutions in Spain [26]. It allows users to send geolocated images of adult mosquitoes of five targeted invasive mosquito species as well as of breeding sites. These data are validated online by experienced Mosquito Alert entomologists, who tag them based on a priori classification criteria and system that associates each image with a likelihood of belonging to a given species [27]. Although the data collection is anonymous, unique participants can be distinguished from one another using randomly assigned Universally Unique Identifiers (UUIDs), which makes it possible to estimate the spatio-temporal variation in sampling effort. Based on the background tracking data, from the launch of the system in June 2014 through the end of 2020, an estimated 432 Mosquito Alert participants spent at least some time in Asturias, with an estimated 279 in Cantabria and 608 in the Basque Country (data not shown). The platform has seen a steady increase in the number of participants over time, with a global acquisition of approximately 124,000 registered participants between June 2014 and June 2021. At the same time, like other citizen science projects, we observe a tendency of participants to engage most with the app when they first install it, and to engage less over time [26]. A strong communication program is in place helping to promote both user acquisition and engagement, which includes an interactive website (http://www.mosquitoalert.com), social media, education activities, community outreach, communication through the app itself and science events. The Mosquito Alert system also makes it possible for vector managers to send messages to specific participants based on their reports or their approximate locations, while maintaining anonymity. From such a notification system, one can send messages either to all devices present in a given area or to a single device. Shortly after the detection of the species by Mosquito Alert in Asturias in June 2018, this tool was exploited regionally to encourage direct reports of *Ae. japonicus* in northern Spain, in combination with synchronous press releases and social media dissemination.
(ii) Regular field monitoring was performed between 2018 and 2020 by the Spanish Government under an agreement between the the Center for Coordination of Alerts and Health Emergencies (CCAES) of the Ministry of Health and the University of Zaragoza. In the National Plan for the Preparedness and Response against Vector-borne Diseases [28] (VBD-NP), targeted sampling of invasive mosquito species is based on egg detection with ovitraps, adult captures and sampling of larval breeding sites. This work completes the routine surveillance at points of entry (PoE) under the International Health Regulations [29] performed by the same team, although no seaports or airports in northern Spain are included. As already described, Mosquito Alert cooperates with the Ministry of Health as a part of the VBD-NP. Thus, detections of invasive mosquito species by Mosquito Alert in new areas have resulted in missions for field confirmation.
(iii) Monitoring of *Culicoides* biting midges (Diptera: Ceratopogonidae), which are vectors of bluetongue virus (BTV), has been performed nation-wide since 2005 by the Ministry of Agriculture (also under contract with the University of Zaragoza) using CDC-UV traps (Miniature Blacklight trap 1212l John W. Hock Company, Gainesville, FL, USA) in cattle farms 1 night per week all year long. These trappings routinely provide Culicidae specimens, which are also identified by morphology and recorded. Although the trapping methods and timings are not specific for invasive *Aedes* mosquito species, farm locations and their associated landscape are indeed a suitable habitat for *Ae. japonicus*. The sampling locations in the study area for the period 2018–2020 included one farm in Asturias, two in Cantabria and one in the Basque Country.
(iv) A regional surveillance program aimed at invasive mosquito species has been conducted since 2013 by the Department of Public Health of the Basque Government and the public agency NEIKER. Surveillance of invasive *Aedes* spp. was based on the deployment of ovitraps in areas with heavy road traffic, both in suburban areas and in the town centers. The number of municipalities surveyed has increased over the years, with a total of 15 municipalities in the province of Bizkaia during 2020 [30]. In addition, routine monitoring for native Culicidae was performed by NEIKER in three sites from the same province using two CDC miniature light traps with incandescent lamp (John W. Hock Company) baited with CO_2_ and deployed for 24 h every 2 weeks between June and October. Potential breeding sites were also checked for immature mosquitoes at these sampling locations. In the study, a fourth municipality was added for larval sampling, accounting for a total of two urban and two rural locations. Also in the Basque Country, an additional separate survey was carried out in 2020 in the province of Araba following the protocols from the AIMCost-Survey (http://www.aedescost.eu/aimsurv) aimed at harmonizing European sampling protocols for invasive mosquito species. A total of 26 ovitraps were deployed by NEIKER in three rural parking lots during 5 months (June to October), and the presence of eggs was checked every 2 weeks.
(v) Sporadic samplings have been performed on the basis of opportunity by some of the authors while traveling across Cantabria for other purposes or during personal leisure time.

In summary, the known sampling efforts in northern Spain in 2018–2020 consisted of (i) citizen scientists sending mosquito reports to Mosquito Alert, (ii) targeted field monitoring by the Spanish Ministry of Health (partly guided by citizen scientists’ findings), (iii) mosquito specimens collected under the entomological surveillance of bluetongue vectors from the Ministry of Agriculture, (iv) a regional monitoring program and (v) personal researcher activity.

## Results

As a result of this multiple-sourced *Ae. japonicus* surveillance deployment from 2018 to 2020 combining active surveillance and citizen science digital sampling tools, we describe here the first findings of *Ae. japonicus* populations in two new autonomous communities of Spain, Cantabria (2019) and all three provinces of the Basque Country (2020). All of the described sampling sources detected *Ae. japonicus* at some time, with the exception of the entomological surveillance of BTV vectors, which provided 58 Culicidae samples between 2018 and 2020 consisting of 54 adult specimens in Asturias, 110 in Cantabria and 11 in Gipuzkoa among which *Ae. japonicus* was not present (data not shown).

**Fig. 2:**
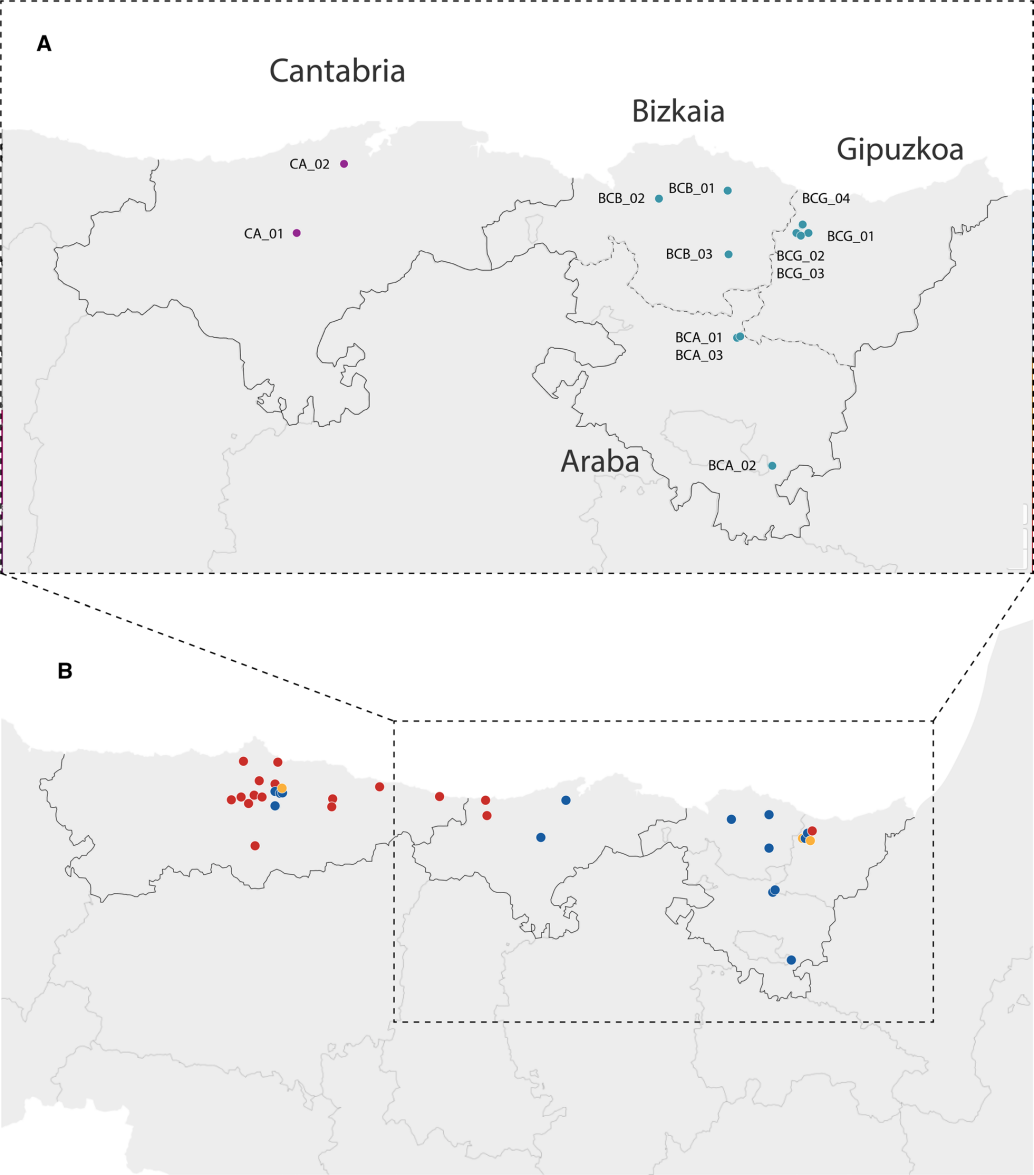
Spatial references of *Ae. japonicus* presence in the Cantabrian cornice (2018–2020). **a** Detailed field findings reported in this work in Cantabria (purple dots) and in the Basque Country (blue dots). Hyphenated line delimits province limits for the Basque Country. **b** Field-confirmed locations (blue dots) include (i) the locations in **a** for Cantabria and the Basque Country (2019–2020), (ii) the 2018 locations in Asturias and (iii) citizen science reports (2017–2020) validated in Mosquito Alert as *Ae. japonicus*, either unverified (red) or field-confirmed (yellow); the respective georeferences are listed in Table 1 (i), in Eritja et al. 2019 [5] (ii) and in Table 2 (iii)

As adult traps did not collect any specimens, all positive samples consisted of eggs from ovitraps, larvae from active checks in breeding sites and a single hand-collected adult. We describe here only positive records that were relevant in the context of the first findings for *Ae. japonicus* in each province or autonomous community. These findings are displayed in Fig. 2, listed in Table 1 and described in the following sections with references to the monitoring context.

**Table 1:** Positive field samplings in the three provinces in the Basque Country Cantabria

Code	Sample source	Autonomous community	Municipality	Coordinates	Date (year/month/day)	Captured stage	Container	Environment
CA_01	MAM (author)	Cantabria	Arenas de Iguña	43.219722°N, 4.055277°W	2019/04/22	14 L1/L2 larvae	Stone trough	Forest
CA_02	IR-A (author)	Cantabria	Escobedo de Camargo	43.385838°N, 3.902975°W	2019/05/02	1 Adult female	N/A	Rural
BCG_01	Mosquito Alert	Basque Country-Gipuzkoa	Elgoibar	43.213947°N, 2.4089284°W	2020/05/16	Image-based	N/A	Suburban
BCG_02	Mosquito Alert	Basque Country-Gipuzkoa	Elgoibar	43.215797°N, 2.418959°W	2020/06/15	Image-based	N/A	Suburban
BCG_03	SD-E (author)	Basque Country-Gipuzkoa	Elgoibar	43.214115°N, 2.408964°W	2020/07/06	Larvae	Drum	Suburban
BCG_04	SD-E (author)	Basque Country-Gipuzkoa	Elgoibar	43.235628°N, 2.400673°W	2020/07/06	Larvae + females	Flower pot	Suburban
BCB_01	NEIKER	Basque Country-Bizkaia	Arratzu	43.320771°N, 2.642686°W	2020/06/22	Larvae	Livestock trough	Rural-farmhouse
BCB_02	NEIKER	Basque Country-Bizkaia	Zamudio	43.302602°N, 2.869214°W	2020/07/08	Larvae	Artificial container	Rural-farmhouse
BCB_03	NEIKER	Basque Country-Bizkaia	Durango	43.167965°N, 2.641472°W	2020/08/10	Eggs	Ovitrap	Suburban parking lot
BCA_01	NEIKER	Basque Country-Araba	Legutiano	42.968823°N, 2.618236°W	2020/06/01	Eggs	Ovitrap	Parking lot-golf course
BCA_02	NEIKER	Basque Country-Araba	Urturi	42.661204°N, 2.509859°W	2020/07/01	Eggs	Ovitrap	Parking lot-golf course
BCA_03	NEIKER	Basque Country-Araba	Legutiano	42.969014°N, 2.618179°W	2020/07/15	Eggs	Ovitrap	Parking lot-golf course

### Cantabria

The first report of *Ae. japonicus* in Cantabria was obtained by one of the authors (MAM) on 22 April 2019 in a rural area of the municipality of Arenas de Iguña (sample code CA_01, Table 1; Fig. 2) while on a family vacation. Sampling the water in an abandoned, traditional stone trough containing abundant organic debris and mosquito larvae (L1–L2) allowed collection of 14 immatures that were raised to adults, subsequently confirmed to be *Ae. japonicus* by morphology and then by genetic barcoding (data not shown). Both the breeding site and the surrounding habitat were similar to those usually described as optimal for the species. Vegetation included mostly oak trees providing shadow and organic debris to water containers in the area. The location was 3800 m distant from the nearest urban area (Bostronizo), and cattle were hypothesized to be the main source for blood-feeding. No other sites were sampled. This finding was officially reported by the author to the Ministry of Health on 3 May 2019. That report was administratively forwarded to the Vectornet network (https://www.ecdc.europa.eu/en/about-us/partnerships-and-networks/disease-and-laboratory-networks/vector-net) to update the European Centre for Disease Prevention and Control (ECDC) map reporting Cantabria as positive on an “introduced” status [25].

On 2 May 2019 another of the authors (IRA), who at the time was unaware of the previous event, made another discovery, also while enjoying leisure time, in the municipality of Escobedo de Camargo (sample code CA_02; Fig. 2), at a straight distance of < 22 km from sample CA_01. In this case, an adult female was captured using a mouth aspirator while it was flying around humans, although not attempting to land on them. This female was also confirmed to be *Ae. japonicus* by DNA barcoding (data not shown). A surrounding forest with grazing cows and horses was examined with no further results. Two larval breeding sites (a bath and a well) within 100 m distance of the capture point were checked and found to contain larvae of *Anopheles*, *Culex* and other *Aedes* species but not *Ae. japonicus*; two other more distant sites (at 1000 and 1500 m from the positive point) were also sampled and resulted negative for any Culicidae species.

### Basque Country

The first report of the presence of *Ae. japonicus* in the Basque Country was obtained in Gipuzkoa province by a citizen sending a report to the Mosquito Alert platform. This report (BCG_01) was received on 16 May 2020 and was geolocated in the municipality of Elgoibar. On 15 June 2020, another citizen scientist sent a second report from a nearby location (BCG_02). Both participants were notified by phone with a request for more information. They responded, agreed to non-anonymous communication and enthusiastically collaborated in collecting larvae. These actions provided all the required information and guided the CCAES coordinated field trip from 6 to 9 July 2020 under the leadership of one of the authors (SDE). That visit resulted in full confirmation of the presence of the species by collecting larvae from a drum in an industrial area in the surroundings of the BCG_01 location (site coded BCG_03) and larvae from a flower pot as well as adults in another nearby location (BCG_04) in a suburban environment. No adult traps were deployed in the context of a one-way fast trip.

Araba Province was determined to be positive for the presence of *Ae. japonicus* on 1 June 2020. A total of three out of the 26 ovitraps located in the parking lots of two golf courses resulted sporadically in the collection of *Ae. japonicus* (codes BCA_01, BCA_02 and BCA_03) (Table 1). All eggs were hatched and emerging adults were morphologically identified as *Ae. japonicus* as well as by DNA barcoding. Sample BCA_02 is at present the southernmost known positive location for *Ae. japonicus* in Spain.

Within the invasive mosquito species surveillance program described for Bizkaia, one of the 15 municipalities investigated was determined to be positive for the presence of *Ae. japonicus* on 10 August 2020, when 18 eggs were found in an ovitrap located in a parking lot of a suburban supermarket in Durango (BCB_03). The eggs were raised to adults and morphologically determined as *Ae. japonicus*. Further ovitraps examined until November 2020 were all negative. Monitoring of native mosquito species in urban and rural areas of Bizkaia using CDC traps did not result in any capture of *Ae. japonicus* adults. However, checking breeding sites resulted, on 22 June 2020 in the collection of fourth-instar larvae from a trough in a sheep farm in Arratzu municipality (BCB_01). Later on, on 8 July 2020, *Ae. japonicus* larvae were also collected from an artificial container at an abandoned farmhouse in Zamudio (BCB_02). Further samplings of breeding sites in these two areas were negative. All immature stages were raised to adults in the laboratory for species confirmation.

### Additional mosquito alert reports

As a result of the official notification of the *Ae. japonicus* finding in 2018, which included a press release, mobile phone notifications and regional outreach, Mosquito Alert received 19 additional reports in northern Spain between 2018 and 2020 whose pictures were validated by experts in two probability levels, either as “Probably *Ae. japonicus*” or “Definitely *Ae. japonicus*”. These reports are listed in Table 2 and shown in Fig. 2; no further confirmation by field sampling was carried out, as at this time participants did not respond back to Mosquito Alert mobile phone notifications, and resources for a proper field campaign were not available. The two first reports in Table 2 were received before the species discovery in 2018 and they were reclassified as “Probably *Ae. japonicus*” during a retrospective revision of the whole northern set of reports in Mosquito Alert, performed in late 2018.

**Table 2:** Reports from the Mosquito Alert database (visible on the public map at www.mosquitoalert.com) sorted by date and marked as “Probably” or “Definitely” *Aedes japonicus* by using predefined criteria

Code	Autonomous community	Municipality	Coordinates	Estimated probability	Date (year/month/day)	UUID
UMA_01	Asturias	Oviedo	43.352047°N, 5.8698854°W	Probably	2017/04/23	adf58f02-ee8f-4766-9a80-5523c8c0ede9
UMA_02	Asturias	Oviedo	43.38453°N, 5.8403°W	Probably	2018/07/15	F00EEF7B-26D8-4594-9FD3-5285484452F2
UMA_03	Asturias	Llanera	43.45552°N, 5.8119764°W	Definitively	2018/08/04	8bbce02f-f3be-4934-aa32-7fafccda9464
UMA_04	Asturias	Siero	43.434643°N, 5.7108703°W	Definitively	2018/08/05	48952eba-73fc-4b59-95fc-f33f57db0b9a
UMA_05	Asturias	Siero	43.38074°N, 5.79261°W	Definitively	2018/08/06	c9503052-5a3f-4701-a44d-31d0b251b6c1
UMA_06	Asturias	Oviedo	43.379036°N, 5.920224°W	Probably	2018/09/30	aa398572-d6bb-4263-9ec8-ba12c067b2a4
UMA_07	Cantabria	Valdáliga	43.315261°N, 4.392075°W	Probably	2018/10/14	8FDAD1FB-ADE4-41B4-90F6-F30CFE6667F1
UMA_08	Asturias	Avilés	43.538498°N, 5.9115167°W	Probably	2019/05/10	a3fd07ba-1a35-49ef-a972-0bfb324b5b06
UMA_09	Asturias	Grado	43.365593°N, 5.980363°W	Definitively	2019/06/03	cb7c28f2-5ce4-4abf-8a39-c3ade981635c
UMA_10	Asturias	Llanes	43.400734°N, 4.686217°W	Probably	2019/07/21	d280e25a-e8b3-44b6-822c-63637d128f48
UMA_11	Asturias	Piloña	43.38124°N, 5.352286°W	Probably	2019/07/22	cb0dcb8e-33d4-494e-bf7c-45a4ac1b7b8f
UMA_12	Asturias	Gijón	43.541695°N, 5.700088°W	Probably	2019/08/22	d4809962-7843-458d-b468-f5dc9e7d0821
UMA_13	Asturias	Piloña	43.346462°N, 5.35454°W	Probably	2019/10/29	8eed95cf-67e4-43da-bf54-de19d3836c49
UMA_14	Asturias	Ribadesella	43.439888°N, 5.061268°W	Probably	2020/07/07	d03cc38b-0372-455b-9609-eeebfd6fba63
UMA_15	Cantabria	S. Vicente de la Barquera	43.379612°N, 4.3997245°W	Probably	2020/08/16	796bd6bb-6db8-4c8c-9f8f-4b4b31f28cad
UMA_16	Basque Country	Mendaro	43.24881°N, 2.3990884°W	Probably	2020/09/05	7932eee1-047f-4b18-a06e-a9ed25c29fdb
UMA_17	Asturias	Lena	43.160428°N, 5.826878°W	Probably	2020/10/18	cf496a31-33d7-4851-9c86-c8aafa5c451b
UMA_18	Basque Country	Elgoibar	43.214854°N, 2.4139035°W	Definitely	2020/10/22	8a7a77b6-7e0e-46e0-9fdf-ff43dfb6140e
UMA_19	Basque Country	Elgoibar	43.2139994°N, 2.413571°W	Probably	2020/10/23	0bdecff6-15a0-4273-929c-3d3f25e635c0

*UUIDS* Universally Unique Identifiers

## Discussion

Getting to know the dispersal strategies of invasive species is fundamental since surveillance programs should be adapted to their ecology of dispersion. It is assumed that invasive mosquitoes use stratified dispersal strategies [31] combining long-distance passive transportation by bulk merchandise carriers, such as boats and trucks, with mid/short-range dispersal by cars and by active flight [32]. Because of this, many invasive mosquito species surveillance programs aim at real-time detection of the arrival of adults at international PoEs, combined with detection of early dispersal by ovitrapping along major roads.

Among PoEs, airports were highly relevant in the past, mostly due to the concern over the arrival of infected *Anopheles* spp. females starting local malaria transmission [33]. Although other species qualifying as invasive arrive at a notable rate in airports as well [34], those low-density introductions do not usually result in local establishment. Nowadays, the major challenge is the bulk introduction of immature stages of Aedine species through commercial shipments connecting different systems of circulation through a globalized trade [35]. The likelihood of establishment this way is much higher compared to arrival at airports, considering the number of individuals per event and the volume and frequency of transport. Additionally, increasing commercial mobility combined with the free circulation of goods within the EU makes it more difficult to trace back introduction routes and establishing quarantines.

Active surveillance by public health agencies is mostly focused on international or regional PoEs because targeting wider hypothesized risk areas is cost-intensive and not scalable, and it needs sound risk assessments to maximize the chance of success. As an alternative, systems promoting minimally-oriented data collection through mobile phones have higher reactivity and much broader spatial and temporal coverage, although as digital data gathered by non-experts, authoritative validation must be performed in the field [26]. These field verifications become a new duty of public health agencies, opening a door for more scalable and flexible surveillance systems that can focus the field efforts on targeted samplings exploiting key contextual data provided by citizens, which often also collaborate in the sampling itself. From this mutually beneficial relationship, citizen-based surveillance programs also benefit from the powerful communication channels available to public health agencies, thereby increasing public awareness, knowledge of invasive mosquito species and engagement with the citizen science platforms. This virtuous circle clearly expands reciprocal communication that educates and empowers the public, potentially promoting societal change and improving surveillance of the invasive mosquito species, as demonstrated by the high level of public cooperation in citizen science programs already in place in the EU [36]. However, in practice this cooperation can be challenging as these programs are often managed by academic institutions with a research focus, whereas public health agencies are of operational nature, with different rhythms and goals.

Multi-sourced surveillance has clearly expanded current knowledge on *Ae. japonicus* distribution. The first detection in Spain in 2018 was not an isolated event but rather a country-wide matter of concern, triggering interest among academics, experts, other citizens and Public Health agencies and leading to new findings in subsequent years in northern Spain. This success in detection of this species by different means in geographically separated locations supports the view that the distribution of *Ae. japonicus* is wider than expected in the Cantabrian cornice. Whereas the field-verified information shows obvious geographical gaps, this assumption is supported by a fair number of geolocated citizen reports, validated by expert entomologists as “Probably” and “Definitely” *Ae. japonicus* pictures, although not verified in the field. Most of these citizen reports came from Asturias, but it is worth noting that one report from Cantabria (coded UMA-07) was received as early as 14 October 2018.

This detection success of a modest species in regions of Spain where no formal surveillance was carried out contrasts with the lack of further administrative reaction for control, mitigation, dispersal limitation or even eradication. Mosquito control operations in the absence of outbreaks of vector-borne diseases are, under Spanish law, the exclusive duty of municipalities. To the best of our knowledge, even after official notification of *Ae. japonicus* presence was made to national and municipal authorities, no control actions were taken in any of the areas where this species was detected. The rural environment where the species was found, with generally low human population density, as well as the low level of nuisance caused by this species, may have contributed to the low response of authorities for the control of *Ae. japonicus*. Moreover, many of the local governments complain that they lack the resources to implement control programs, even in some regions where *Ae. albopictus* is already present. In contrast, in some large cities (e.g. Barcelona, Valencia) multi-sourced strategies show all their potential to manage urban *Aedes* species, such as the Asian Tiger mosquito, as digital citizen science is starting to be integrated into the control protocols as an additional tool, allowing not only the use of citizen contributions in incidence maps but also the modeling of vector exposure risk from an epidemiological point of view.

The sequence and dynamics of the colonization process in the Cantabrian cornice are therefore uncertain. Nonetheless, whereas in 2018 the range estimated by building a polygonal area from the outermost points had an area of 827 km^2^, these new data shift the probable colonized area in northern Spain to > 7000 km^2^.

In terms of habitat, it is worth noting that *Ae. japonicus* was found in a variety of breeding sites, ranging from cattle troughs in farms and forested areas, to drums and flower pots in suburban areas. Despite the fact that this species is not considered a high-risk mosquito for disease transmission, its future role is unknown if colonization of rural and/or urban areas expands in both abundance and distribution.

Sampling *Aedes japonicus* using standard field sampling methods is challenging [37], in particular when using adult traps (i.e. CDC and BG-Sentinel traps) routinely used for other invasive species, such as *Ae. albopictus* and *Ae. aegypti* [22]. Most of the detections presented here were based on larval collection and egg-laying in ovitraps. Adults were only captured in one case and not by trapping but by aspiration. This low sensitivity of the species to the common sampling tools also highlights the usefulness of combining field sampling and citizen-sourced data, which resulted in 19 reports (with photographs) during the same period.

The introduction routes of *Ae. japonicus* to Spain are unknown. The used tire trade is commonly assumed to be the main driver of invasive mosquito species introduction, and ground transportation in cars to be a key determinant for further spreading [34]. However, a general relationship between *Ae. japonicus* invasions and the tire trade is unclear, as to date only two cases in France and Belgium could be linked to the commercial tire trade, and a tire depot found to be infested in the Netherlands in 2013 was probably a secondary colonization [19]. In other cases, shipments of cemetery plants from Asia [16] or Dutch greenhouse imports [34] have also been identified as the likely sources. Unlike other invasive species, a relationship between spreading and road transport is not obvious for *Ae. japonicus* [38], and its introduction to many EU countries is currently explained as “natural” or “unknown” [34]. A scarcity of data points in Spain do not allow the possibility of a dispersal relationship with road networks to be assessed.

No regular surveillance on the arrival of *Ae. japonicus* had ever been envisaged in Spain, as its landing was considered to be a highly unlikely event. In 2018 the nearest known European population was as far away as 1100 km, in north-eastern France. Moreover, an intense surveillance campaign focused on *Ae. albopictus*, which was carried out on French roads for several years up to 2017, had not recorded any *Ae. japonicus* specimens [39]. The lack of evidence for the presence of *Ae. japonicus* in any region between northern Spain and north-eastern France suggests an overseas introduction to Spain, or perhaps a single long-distance road event, followed by spread across rural areas of the Cantabrian cornice using roads and/or autonomous dispersal through natural corridors.

Autonomous flight has been suggested as the main driver for the 100-km expansion in dispersal range in Austria over a 7-year period, as well as its expansion in Hungary [23, 40] and its spread of up to 250 km in Croatia in only 5 years [22]. However, the role of active flight abilities is unclear. In a literature survey assessing the average maximum flight distance of Culicidae, a mean distance of 676 m for *Ae. albopictus* was considered as low, especially when compared to other long-range species such as *Ae. vexans* (5727 m) [41]. In that study, *Ae. japonicus* was qualified as being of moderate capacity in terms of flight distance, with an average maximum distance of 1600 m. It has been suggested that its natural dispersal could be facilitated by riparian corridors rather than road corridors, since this species often colonizes rock pools [42], accounting for a silent establishment in remote natural areas from which it could spread to peri-urban areas. These active dispersal schemes are plausible as *Ae. japonicus* tolerates low temperatures, thus allowing upstream dispersal to highland locations [42].

Is the *Ae. japonicus* population detected thus far in Spain the tip of an iceberg? Assessment of non-native populations is complex, especially if population density is low—or is just perceived as such, secondary to the reduced level of anthropophily. Just as the tip of an iceberg is misleading in regards to the real size of the object, these new locations describe, as hypothesized, a much broader presence of the species than the original discovery area in 2018. Just like an icebergs’ total size, in order to reliably assess the real dimensions it will take extensive diving into the problem as it is not possible at the initial stages to distinguish early detection of a newly established population from late detection of a low-density but long-established population, usually defined as a sleeper population [43]. The sleeper concept suggests that many non-native species are more widespread than one would expect a priori because they can maintain populations at low numbers, producing non-measurable impacts and thereby rarely becoming noticed [44]. Nevertheless, the sleeper species are present in the system and can spread silently, having the potential to produce population outbreaks of high impact, if triggered by key environmental factors. In fact, ecological niche models clearly indicate that the potential distribution of *Ae. japonicus* in non-native areas such as North America and Europe is underestimated, therefore suggesting a silent spread in those areas [45].

Silent and unnoticed large-scale colonization is not uncommon for this species in Europe [1, 16]. It is remarkable how a mammalophilic, large-bodied conspicuous species as *Ae. japonicus* can settle over such large areas and be noticed by only a few citizen scientists. As an example of the opposite phenomenon, some urban species, such as *Culiseta longiareolata*, are frequently reported by Mosquito Alert participants, despite of their strict ornithophilic behavior. The low nuisance level caused by *Ae. japonicus* in rural areas contrasts with the social stress raised by *Ae. albopictus* which has a more aggressive behavior and is linked to an urban habitat. Therefore, factors driving citizen scientists’ propensity for reporting a mosquito appear to be related with the body size, behavioral aggressiveness and its presence in densely populated areas.

At least four *Ae. japonicus* population clusters are considered at the European level and a minimum of two separate introductions are suspected on the basis of genetic structuration [14], with additional isolated haplotypes referenced in Croatia [22].

## Conclusions

The distribution, isolation and size of the *Ae. japonicus* cluster detected in northern Spain could be the result of overseas introduction, and this possibility merits additional work, especially in terms of sampling in the neighboring region of Galicia in the same climate area. It is likely such a sampling effort would show a broader distribution than what is currently known. In the coming years we aim to assess the genetic relationships among the EU clusters and compare those to *Ae. japonicus* populations in other regions of the world in order to infer dispersal strategies. The high value of multi-source strategies in combination with field sampling by public health agencies and reports from citizen scientists and academia makes for a powerful surveillance tool. These strategies can be implemented not only for detection of invasive mosquito species at a local, national and supranational level, but also for control programs against already established species. The major vector species *Aedes aegypti* has already been reported from some areas of Europe, and climatic conditions may well enable its establishment across southern Europe, which would greatly increase the risk of transmission of the chikungunya, dengue, yellow fever and Zika viruses. As demonstrated by the findings by Mosquito Alert of new invasive mosquito species and locations in Spain, citizen science makes it possible to broaden temporal and spatial context data, resulting in a more flexible and more cost-effective invasive mosquito species sampling strategy.

## Data Availability

All data supporting the conclusions of this article are provided within the article. All citizen reports are available in the public map at http://www.mosquitoalert.com.

## Abbreviations

CCAES: Spanish Coordination Center for Health Alerts and Emergency Events
ECDC: European Centre for Disease Prevention and Control
NUTS: Nomenclature of Territorial Units for Statistics
PoE: Point of entry
VBD-NP: Spanish National Plan for the Preparedness and Response against Vector-borne Diseases

## References

[1] Schaffner F, Kaufmann C, Hegglin D, Mathis A (2009). The invasive mosquito *Aedes japonicus* in Central Europe. Med Vet Entomol.

[2] Bartlett-Healy K, Unlu I, Obenauer P, Hughes T, Healy S, Crepeau T (2012). Larval mosquito habitat utilization and community dynamics of *Aedes albopictus* and *Aedes japonicus* (Diptera: Culicidae). J Med Entomol.

[3] Cunze S, Koch LK, Kochmann J, Klimpel S (2016). *Aedes albopictus* and *Aedes japonicus*—two invasive mosquito species with different temperature niches in Europe. Parasites Vectors..

[4] Huber K, Pluskota B, Jöst A, Hoffmann K, Becker N (2012). Status of the invasive species *Aedes japonicus japonicus* (Diptera: Culicidae) in southwest Germany in 2011. J Vector Ecol.

[5] Eritja R, Ruiz-Arrondo I, Delacour-Estrella S, Schaffner F, Álvarez-Chachero J, Bengoa M (2019). First detection of *Aedes japonicus* in Spain: an unexpected finding triggered by citizen science. Parasites Vectors.

[6] Cebrián-Camisón S, Martínez-De La Puente J, Figuerola J (2020). A literature review of host feeding patterns of invasive *Aedes* mosquitoes in Europe. Insects..

[7] Schaffner F, Chouin S, Guilloteau J (2003). First record of *Ochlerotatus* (*Finlaya*) *japonicus japonicus* (Theobald, 1901) in metropolitan France. J Am Mosq Control Assoc.

[8] Kampen H, Werner D (2014). Out of the bush: The Asian bush mosquito *Aedes japonicus japonicus* (Theobald, 1901) (Diptera, Culicidae) becomes invasive. Parasites Vectors.

[9] Turell MJ, Dohm DJ, Sardelis MR, O’Guinn ML, Andreadis TG, Blow JA (2005). An update on the potential of North American mosquitoes (Diptera: Culicidae) to transmit West Nile virus. J Med Entomol.

[10] Harris MC, Dotseth EJ, Jackson BT, Zink SD, Marek PE, Kramer LD (2015). La Crosse Virus in *Aedes japonicus japonicus* mosquitoes in the Appalachian region, United States.. Emerg Infect Dis..

[11] Wagner S, Mathis A, Schönenberger AC, Becker S, Schmidt-Chanasit J, Silaghi C (2018). Vector competence of field populations of the mosquito species *Aedes japonicus japonicus* and *Culex pipiens* from Switzerland for two West Nile virus strains. Med Vet Entomol.

[12] Schaffner F, Vazeille M, Kaufmann C, Failloux AB, Mathis A (2011). Vector competence of *Aedes japonicus* for chikungunya and dengue viruses. Eur Mosq Bull.

[13] Takashima I, Rosen L (1989). Horizontal and vertical transmission of Japanese encephalitis virus by *Aedes japonicus* (Diptera: Culicidae). J Med Entomol.

[14] Koban MB, Kampen H, Scheuch DE, Frueh L, Kuhlisch C, Janssen N (2019). The Asian bush mosquito *Aedes japonicus japonicus* (Diptera: Culicidae) in Europe, 17 years after its first detection, with a focus on monitoring methods. Parasites Vectors.

[15] Versteirt V, Schaffner F, Garros C, Dekoninck W, Coosemans M, Van Bortel W (2009). Introduction and establishment of the exotic mosquito species *Aedes japonicus japonicus* (Diptera: Culicidae) in Belgium. J Med Entomol.

[16] Becker N, Huber K, Pluskota B, Kaiser A (2011). *Ochlerotatus japonicus japonicus*—a newly established neozoan in Germany and a revised list of the German mosquito fauna. Eur Mosq Bull.

[17] Kampen H, Kuhlisch C, Fröhlich A, Scheuch DE, Walther D (2016). Occurrence and spread of the invasive asian bush mosquito *Aedes japonicus japonicus* (diptera: Culicidae) in west and north Germany since detection in 2012 and 2013, respectively. PLoS ONE.

[18] Seidel B, Montarsi F, Huemer HP, Indra A, Capelli G, Allerberger F (2016). First record of the Asian bush mosquito, *Aedes japonicus japonicus*, in Italy: Invasion from an established Austrian population. Parasites Vectors.

[19] Ibañez-Justicia A, Kampen H, Braks M, Schaffner F, Steeghs M, Werner D (2014). First report of established population of *Aedes japonicus japonicus* (Theobald, 1901) (Diptera, Culicidae) in the Netherlands. J Eur Mosq Control Assoc.

[20] Krebs T, Bindler P, L’Ambert G, Toty C, Perrin Y, Jourdain F (2014). First establishment of *Aedes japonicus japonicus* (Theobald, 1901) (Diptera: Culicidae) in France in 2013 and its impact on public health. J Vector Ecol.

[21] Klobučar A, Lipovac I, Žagar N, Mitrović-Hamzić S, Tešić V, Vilibić-Čavlek T (2019). First record and spreading of the invasive mosquito *Aedes japonicus japonicus* (Theobald, 1901) in Croatia. Med Vet Entomol.

[22] Janssen N, Graovac N, Vignjević G, Sudarić Bogojević M, Turić N, Klobučar A (2020). Rapid spread and population genetics of *Aedes japonicus japonicus* (Diptera: Culicidae) in southeastern Europe (Croatia, Bosnia and Herzegovina, Serbia). PLoS ONE..

[23] Seidel B, Nowotny N, Bakonyi T, Allerberger F, Schaffner F (2016). Spread of *Aedes japonicus japonicus* (Theobald, 1901) in Austria, 2011–2015, and first records of the subspecies for Hungary, 2012, and the principality of Liechtenstein, 2015. Parasites Vectors.

[24] Horváth C, Cazan CD, Mihalca AD (2021). Emergence of the invasive Asian bush mosquito in an urban area. Romania. Parasites Vectors..

[25] European Centre for Disease Prevention and Control and European Food Safety Authority. Mosquito maps. Stockholm. 2020. https://ecdc.europa.eu/en/disease-vectors/surveillance-and-disease-data/mosquito-maps. Accessed 17 Jan 2021.

[26] Palmer JRB, Oltra A, Collantes F, Delgado JA, Lucientes J, Delacour S (2017). Citizen science provides a reliable and scalable tool to track disease-carrying mosquitoes. Nat Commun..

[27] Oltra, A, Palmer J, Bartumeus F. Atrapaeltigre.com: Enlisting citizen-scientists in the war on tiger mosquitoes. In: Capineri C, Haklay M, Huang H, Antoniou V, Kettunen J, Ostermann F, et al., editors. European handbook of crowdsourced geographic information. London: Ubiquity Press; 2016. p. 295–308. http://www.ubiquitypress.com/site/books/detail/28/european-handbook-of-crowdsourced-geographic-information/

[28] Andradas Aragonés E (editor). Plan Nacional de preparación y respuesta frente a enfermedades transmitidas por vectores, Parte I: Dengue, Chikungunya y Zika. 2016. http://www.mscbs.gob.es/profesionales/saludPublica/ccayes/alertasActual/DocsZika/Plan_Nac_enf_vectores_20160720_sin_CC.pdf. Accessed 3 Dec 2020.

[29] Anonymous. Resumen de los resultados del proyecto “Vigilancia entomológica en aeropuertos y puertos frente a vectores importados de enfermedades infecciosas exóticas, y vigilancia de potenciales vectores autóctonos de dichas enfermedades”. 2016. https://www.mscbs.gob.es/profesionales/saludPublica/ccayes/activPreparacionRespuesta/doc/Resumen_Proyecto_vigilancia_entomologica_2016.pdf. Accessed 10 Jan 2021.

[30] Goiri F, González MA, Goikolea J, Oribe M, de Castro V, Delacour S (2020). Progressive invasion of *Aedes albopictus* in Northern Spain in the period 2013–2018 and a possible association with the increase in insect bites. Int J Environ Res Public Health..

[31] Liebhold AM, Tobin PC (2010). Exploiting the Achilles heels of pest invasions: Allee effects, stratified dispersal and management of forest insect establishment and spread. New Zeal J For Sci..

[32] Eritja R, Palmer JRB, Roiz D, Sanpera-Calbet I, Bartumeus F (2017). Direct evidence of adult *Aedes albopictus* dispersal by car. Sci Rep.

[33] Gratz NG, Steffen R, Cocksedge W (2000). Why aircraft disinfection?. Bull World Health Organ.

[34] Ibáñez-Justicia A (2020). Pathways for introduction and dispersal of invasive *Aedes* mosquito species in Europe: a review. J Eur Mosq Control Assoc.

[35] Lounibos LP (2002). Invasions by insect vectors of human disease. Annu Rev Entomol..

[36] Kampen H, Medlock JM, Vaux AGC, Koenraadt CJM, van Vliet AJH, Bartumeus F (2015). Approaches to passive mosquito surveillance in the EU. Parasites Vectors..

[37] European Centre for Disease Prevention and Control. Guidelines for the surveillance of invasive mosquitoes in Europe. Stockholm. 2012. www.ecdc.europa.eu. Acccessed 11 Dec 2020. 22971331

[38] Müller P, Engeler L, Vavassori L, Suter T, Guidi V, Gschwind M (2020). Surveillance of invasive *Aedes* mosquitoes along Swiss traffic axes reveals different dispersal modes for *Aedes albopictus* and *Ae. japonicus*. PLoS Negl Trop Dis..

[39] EID Méditerranée. Surveillance du moustique *Aedes albopictus* en France métropolitaine—Bilan 2016. 2016. https://solidarites-sante.gouv.fr/IMG/pdf/bilan_surv_albopictus_2016.pdf. Accessed 3 Oct 2020.

[40] Sáringer-Kenyeres M, Bauer N, Kenyeres Z (2020). Active dispersion, habitat requirements and human biting behaviour of the invasive mosquito *Aedes japonicus japonicus* (Theobald, 1901) in Hungary. Parasitol Res..

[41] Verdonschot PFM, Besse-Lototskaya AA (2014). Flight distance of mosquitoes (Culicidae): a metadata analysis to support the management of barrier zones around rewetted and newly constructed wetlands. Limnologica..

[42] Bevins SN (2007). Establishment and abundance of a recently introduced mosquito species *Ochlerotatus japonicus* (Diptera: Culicidae) in the Southern Appalachians, USA. J Med Entomol.

[43] Frank SD, Just MG (2020). Can cities activate sleeper species and predict future forest pests? A case study of scale Insects. Insects..

[44] Spear MJ, Walsh JR, Ricciardi A, Zanden MJ (2021). The invasion ecology of sleeper populations: prevalence, persistence, and abrupt shifts. Bioscience..

[45] Cunze S, Kochmann J, Klimpel S (2020). Global occurrence data improve potential distribution models for *Aedes japonicus japonicus* in non-native regions. Pest Manag Sci.

